# Quorum Sensing and Quorum Quenching in Pseudomonas aeruginosa and Staphylococcus aureus Infections: Therapeutic Potential, Limitations and Clinical Challenges

**DOI:** 10.3390/antibiotics15050447

**Published:** 2026-04-29

**Authors:** Emilia Nowak, Matylda Korgiel, Karolina Pawłuszkiewicz, Jarosław Widelski, Bachar Cheaib, Emil Paluch

**Affiliations:** 1 Billennium Spółka Akcyjna, Tadeusza Czackiego 15/17, 00-043 Warsaw, Poland; emilia.nowak@billennium.com; 2 Faculty of Medicine, Wroclaw Medical University, 50-376 Wroclaw, Poland; matylda.korgiel@student.umw.edu.pl (M.K.); karolina.pawluszkiewicz@student.umw.edu.pl (K.P.); 3 Department of Pharmacognosy with Medicinal Plants Garden, Lublin Medical University, 20-093 Lublin, Poland; jaroslaw.widelski@umlub.pl; 4 German Center for Lung Research (DZL), Translational Lung Research Center Heidelberg (TLRC), Department of Infectious Diseases, Medical Microbiology and Hygiene, Medical Faculty Heidelberg University, Im Neuenheimer Feld 672, 69120 Heidelberg, Germany; bachar.cheaib@med.uni-heidelberg.de; 5 Department of Microbiology, Faculty of Medicine, Wroclaw Medical University, Tytusa Chalubinskiego 4, 50-376 Wroclaw, Poland

**Keywords:** Quorum sensing, Quorum quenching, biofilms, *Pseudomonas aeruginosa*, *Staphylococcus aureus*, anti-virulence therapy, drug resistance, microbial

## Abstract

Antimicrobial resistance (AMR) represents a major global health threat, largely driven by antibiotic overuse and the protective role of bacterial biofilms. Quorum sensing (QS), a bacterial communication system regulating virulence and biofilm formation, has emerged as a promising therapeutic target. Quorum quenching (QQ), which disrupts QS without directly inhibiting bacterial growth, is considered a potential anti-virulence strategy that may reduce selective pressure for resistance. This review critically evaluates recent advances in QQ research, focusing on its clinical applicability, limitations, and risks. We analyzed studies from the last five years involving natural compounds, synthetic molecules, nanoparticles (NPs), and combination therapies targeting key pathogens such as *Pseudomonas aeruginosa* and *Staphylococcus aureus* in models of lung diseases, mainly cystic fibrosis, chronic wounds, burns, and implant-associated infections. While numerous compounds demonstrate significant in vitro anti-biofilm and anti-virulence activity, major challenges remain, including limited in vivo validation, pharmacokinetic constraints, toxicity concerns, microbiome disruption, and the potential development of tolerance or functional resistance. Although QQ offers a promising adjunctive approach to conventional antibiotics, its long-term clinical feasibility requires comprehensive evaluation of evolutionary dynamics, host–microbe interactions, and safety profiles.

## 1. Introduction

Antimicrobial resistance (AMR), particularly bacterial AMR, is now recognized as one of the most serious global health threats, significantly reducing the effectiveness of infection treatment and prevention strategies. Important insight into the scale of this challenge has been provided by large-scale quantitative analyses, including a systematic study by the Antimicrobial Resistance Collaborators [1], which estimated that AMR accounted for 1.14 million direct deaths in 2021 and may cause 1.91 million deaths annually by 2050 if effective interventions are not implemented [2,3,4].

Table 1 [5] presents the World Health Organisation (WHO) Bacterial Priority Pathogens List (BPPL), including the classification of pathogens into critical, high-, and medium-priority groups, to guide research and development efforts as well as public health interventions. The 2024 WHO BPPL covers 24 pathogens, spanning 15 families of antibiotic-resistant bacterial pathogens. The critical priority category is reserved for pathogens exhibiting resistance to last-resort treatment options, which are distinguished by their high potential for resistance gene transfer, the severity of associated infections, and their substantial global health burden [5].

This challenge is further exacerbated by the predominant mode of bacterial growth in the host ecosystem, where microorganisms rarely persist as planktonic cells, which are highly susceptible to adverse conditions, but instead tend to form biofilms, i.e., highly organized microbial assemblies enclosed within a self-generated extracellular polymeric substance (EPS) matrix that functions as a protective barrier [6]. In general, biofilms are composed of approximately 10–25% microbial cells and 75–90% EPS, although the exact composition of the matrix may differ depending on the microorganisms involved [7,8].

Biofilms play a pivotal role in amplifying antimicrobial resistance through multiple protective mechanisms, serving as hotspots for genetic exchange and environmental persistence [9]. A high degree of heterogeneity within the biofilm promotes the formation of persister cells—metabolically inactive cells that lack transcription, translation, and proton motive force [10,11]. Biofilms are widely acknowledged as key etiological factors in numerous infections, contributing to approximately 65% to 80% of cases [12]. Their prevalence in medical implants, living and dead tissues makes the infections they cause difficult and costly to treat [13]. Biofilm formation constitutes a critical survival strategy for bacteria and significantly contributes to their virulence. The reduced efficacy of antimicrobial therapy in biofilm-associated infections results from multiple interacting factors that enhance bacterial tolerance and resistance to antibiotics. Consequently, conventional monotherapeutic approaches are often insufficient, underscoring the need for the development of alternative and complementary therapeutic strategies. Promising approaches include bacteriophage-based therapies, combination treatments integrating antibiotics with biofilm-disrupting agents or efflux pump inhibitors, and strategies targeting bacterial communication systems, such as quorum sensing (QS) inhibition through quorum quenching (QQ) [1,9,14].

Because the transition from planktonic cells to a highly structured biofilm is strictly governed by QS-dependent gene expression, this communication network is the primary driver of biofilm maturation. QQ directly counteracts this process by degrading or competitively blocking the signalling molecules required for such coordination. Consequently, the application of QQ effectively halts biofilm formation and destabilizes existing structures, stripping pathogens of their protective shield and restoring their susceptibility to conventional antimicrobials [15].

This review summarizes current knowledge on QS systems and QQ mechanisms as a conceptual basis for the analysis of anti-virulence and anti-biofilm strategies. Particular attention is given to natural QQ compounds, synthetic molecules, nanoparticle (NPs)-based platforms, and combination approaches investigated against clinically relevant biofilm-forming pathogens. The discussion focuses primarily on *Pseudomonas aeruginosa* and *Staphylococcus aureus*, which serve as the principal models for evaluating the therapeutic potential, translational limitations, and clinical relevance of QQ-based interventions. Across the manuscript, emphasis is placed not only on synthesizing available evidence, but also on highlighting emerging concepts and potentially valuable future research directions in this field. Overall, this approach is intended to provide both a structured synthesis of current evidence and a broader perspective on promising translational and research avenues for the future development of QQ-based therapies.

## 2. Quorum Sensing (QS) and Quorum Quenching (QQ)

### 2.1. Quorum Sensing (QS) Overview

QS is a signalling mechanism that enables microorganisms to adapt to environmental conditions and regulate gene expression in a coordinated manner through small diffusible molecules—Autoinducers (AIs) [16]. AI quantity rises with the increasing population of bacterial cells. These signalling molecules accumulate in the surrounding environment and, once a critical threshold concentration is reached, bind to specific receptor proteins, triggering coordinated changes in gene expression. Through this strategy, many pathogenic bacteria maintain the adaptation to changing environmental conditions by regulating genes involved in biofilm formation, virulence factor production, antibiotic synthesis, and horizontal gene transfer processes such as transformation and conjugation [15].

Recently, the mechanism of QS has been extensively studied due to its technological and medical applications including combating microbial pathogenicity, biofilm-related infections, and abiotic factors and conditions.

### 2.2. Main QS Systems in Pseudomonas aeruginosa

**Figure 1:**
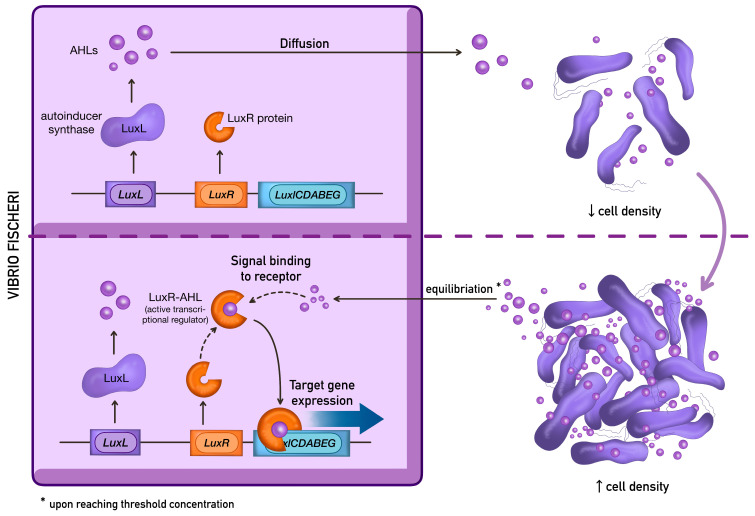
Acyl-homoserine lactone (AHL)-dependent LuxI/LuxR quorum sensing (QS) pathway in *Vibrio fischeri* as a species-specific model of Gram-negative bacterial signalling. At low cell density, the autoinducer (AI) N-(3-oxohexanoyl)-L-homoserine lactone (3OC6-HSL; purple dots), synthesized by LuxI, passively diffuses across the cell membrane into the extracellular environment. Under these conditions, expression of the luxICDABEG operon is minimal. As the bacterial population increases, 3OC6-HSL accumulates in the surrounding medium and, upon reaching a threshold concentration, equilibrates across the membrane. Intracellularly, the AI binds to the transcriptional regulator LuxR, forming an active LuxR–AHL complex, which induces transcription of the luxICDABEG operon. This activation triggers the bioluminescence system, resulting in a marked increase in light emission. This model represents a canonical LuxI/LuxR QS system; however, QS system architecture may vary among different Gram-negative bacteria.

In Gram-negative bacteria, QS is predominantly mediated by acyl-homoserine lactones (AHLs), classified as AI-1 autoinducers. Figure 1 [17] presents the LuxI/LuxR system, which is considered the canonical AHL-dependent quorum-sensing mechanism in Gram-negative bacteria that was first described in *Vibrio fischeri* [17].

Homologues of the LuxI/LuxR-type system have been described in several other Gram-negative bacteria. In *P. aeruginosa*, however, QS is organized into a more elaborate regulatory network composed of four systems. Among them, LasI/LasR and RhlI/RhlR represent two AHL-dependent signalling circuits that direct the synthesis of N-(3-oxo-dodecanoyl)-L-homoserine lactone (3OC12-HSL) and N-butanoyl-L-homoserine lactone (C4-AHL), respectively [18,19,20]. Beyond these canonical AHL-based modules, *P. aeruginosa* also employs the non-AHL Pseudomonas quinolone signal (PQS) system and the more recently characterized integrated quorum-sensing signal (IQS) system [21]. Las and Rhl are LuxI/LuxR systems, in which AHLs—3OC12-HSL and C4-AHL—produced by cognate LuxI-type synthases bind and stabilize LuxR-type transcriptional regulators, enabling DNA binding. Subsequently, the LuxR–AHL complexes interact with specific short DNA sequences named lux-boxes, thereby modulating gene expression. Regarding virulence, the Las system controls the production of several proteases, elastase, the siderophores pyoverdine and pyochelin and exotoxin A [22]. The Rhl system mainly regulates the synthesis of rhamnolipids and pyocyanin (PYO) [23]. The third QS system, PQS, is associated with a 2-alkyl-4-(1H)-quinolone (AHQ) and its precursor, 2-heptyl-4-(1H)-quinolone (HHQ). Both molecules activate the transcriptional regulator PqsR enhancing the expression of the pqsABCDE operon, which triggers the production of several virulence factors (mainly PYO and lectins) and biofilm formation. Finally, the fourth putative QS system, IQS, has been described to integrate QS and stress response. Thus, it has been reported that IQS partially takes over the central role of the Las system under phosphate depletion stress conditions [24]. To provide a structured overview of the contribution of quorum sensing to *P. aeruginosa* pathogenicity, the main QS-regulated virulence determinants are categorized by function in Table 2 [25,26,27,28,29,30].

The significance of QS in human diseases associated with *P. aeruginosa* has been well established. According to numerous investigations, 90% of *P. aeruginosa* samples that can cause infections have working HSL systems. In one instance, it has been demonstrated that patients with cystic fibrosis (CF) typically have N-(3-oxododecanoyl)-HSL, the major *P. aeruginosa* AIs. Polysaccharide alginate, a crucial part of the matrix created by *P. aeruginosa* strains, is a QS-controlled virulence factor that shields biofilms from macrophage destruction [32].

### 2.3. Main QS Systems in Staphylococcus aureus

*S. aureus* infections are particularly problematic due to frequently occurring antibiotic resistance in *S. aureus* isolates, among which methicillin-resistant *S. aureus* (MRSA) are the most important clinically [33].

**Figure 2:**
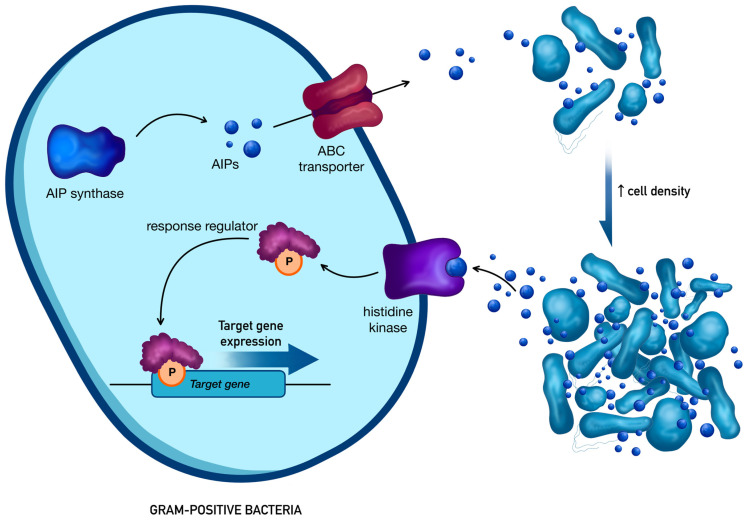
Autoinducing peptides (AIP)-dependent quorum-sensing (QS) signalling pathway in Gram-positive bacteria [34,35]. Similarly to acyl-homoserine lactone (AHL)-based QS systems, the extracellular concentration of secreted AIP autoinducers rises as bacterial cell density increases. Upon activation, the phosphorylated response regulators function as deoxyribonucleic acid (DNA)-binding transcription factors that control the expression of specific target genes. In some Gram-positive bacteria, such as the agr QS system of *Staphylococcus aureus*, the genes encoding the autoinducer precursor, the histidine kinase receptor, and the cognate regulatory protein are organized within a single operon, whose transcription is itself induced by QS. This arrangement establishes a positive feedback loop and promotes a rapid shift from low-cell-density (LCD) to high-cell-density (HCD) quorum-dependent gene expression.

In Gram-positive bacteria, QS is predominantly based on autoinducing peptides (AIPs) derived from the processing of larger precursor proteins. Figure 2 presents the characteristic architecture of a Gram-positive AIP-dependent QS system, which typically includes a membrane-bound histidine kinase receptor and a cognate cytoplasmic response regulator acting at the transcriptional level [34,35].

In *S. aureus*, the agr locus represents the best-characterized AIP-based quorum-sensing system. It operates through two promoters, P2 and P3, which direct the synthesis of ribonucleic acid (RNA) II and RNAIII. The RNAII transcript encodes four essential elements: AgrD, the precursor peptide [36], AgrB, responsible for its maturation and export [36], AgrC, the sensor histidine kinase [37], and AgrA, the associated response regulator [38]. After processing by AgrB, the mature cyclic thiolactone-containing peptide is released outside the cell, where it accumulates as the bacterial population expands. Once sufficient extracellular levels are reached, it is recognized by AgrC, resulting in receptor autophosphorylation [39] and subsequent activation of AgrA. Phosphorylated AgrA increases transcription from both agr promoters, strengthening the system through positive feedback, and also promotes RNAIII production from P3 [40]. RNAIII then acts as the principal downstream effector, controlling the expression of numerous virulence-associated factors, including leukocidins, hemolysins, toxic shock syndrome toxin, exoproteases, and lipases.

Beyond the *agr* system, *S. aureus* also possesses the luxS/AI-2 pathway [41,42]. This pathway, based on AI-2 generated through LuxS-dependent synthesis of the precursor 4,5-dihydroxy-2,3-pentanedione (DPD), has been implicated mainly in the regulation of biofilm formation, rather than in direct control of classical virulence factors [41,42,43,44,45]. Available data indicate that luxS/AI-2 signalling influences the expression of biofilm-associated regulators, including *icaR*, *icaA*, and *rbf*, thereby modulating PIA-dependent biofilm development [41,42,43,44,45].

### 2.4. Role of QQ and Its Mechanisms

The persistence of biofilms and the growing problem of antibiotic resistance highlight the need for alternative antimicrobial strategies. One promising approach involves interference with QS, the regulatory system that coordinates microbial communication. Among such strategies, QQ has attracted particular attention, as it disrupts signalling between microbial cells and can reduce or even completely suppress the production of virulence-associated factors, including those involved in biofilm formation [46]. QQ has evolved to control QS processes by obstructing their key steps, such as:

- Inhibition of signal molecules synthesis;
- Inactivation or enzymatic degradation of signal molecules;
- Competing with signal receptor analogues;
- Blocking the signal transduction cascades.

Unlike antibiotics, which kill bacteria, the QQ process disrupts bacterial communication without damaging the colony, thereby helping to prevent the development of antibacterial resistance. Research into QS and QQ holds significant implications for biotechnology, medicine, and environmental management [6,47].

QQ can interfere with bacterial communication through several mechanisms. The most common involves the enzymatic inactivation of AHL signalling molecules, mediated by enzymes such as lactonases, acylases, reductases, and oxidases. Other strategies include the use of inducer antagonists that block receptor binding, inhibition of signal molecule synthesis (e.g., by interfering with LuxI activity or through kinase inhibitors in Gram-positive bacteria), and disruption of signal transduction pathways, such as inhibition of the AgrA regulator, which ultimately reduces the expression of QS-controlled virulence factors [6].

QQ-active compounds can significantly reduce or even completely inhibit the expression of virulence factors, including biofilm development. Several QQ strategies involve the use of structural analogues of QS AIs, which may occur naturally or be designed and synthesized using chemical engineering techniques. In addition, many well-characterized QQ agents are enzymes capable of degrading signalling molecules [6]. These properties make QQ an increasingly attractive anti-virulence strategy against biofilm-associated infections caused by major bacterial pathogens. At the same time, its successful therapeutic development requires careful recognition of both its potential and its limitations, as well as further research to clarify its efficacy, safety, and long-term clinical applicability.

## 3. QQ Compounds: Current Research Trends

Table 3, Table 4, Table 5 and Table 6 aim to systematize the current evidence on QQ strategies by distinguishing between natural and synthetic compounds evaluated predominantly in *Pseudomonas aeruginosa* and *Staphylococcus aureus* models.

In Table 3, Table 4, Table 5 and Table 6, we also included QQ–antibiotic combinations. A few reports are suggesting that QQ alone may be insufficient for biofilm eradication; instead, combinatorial therapy using QQ with other antimicrobial agents may be more effective [48]. Because of these predictions many authors [10,49,50,51,52], while testing the effectiveness of new QQ molecules, were also checking their potential synergistic effect with antibiotics that have been known for years. As the QS inhibiting strategy relies on attenuation of pathogenicity of infectious bacteria rather than affecting their growth, the possibility of selection pressure and the emergence of resistance is minimized, thereby increasing the susceptibility of pathogens to the host immune system [53].

This comparison was intended not only to identify the most promising QQ strategies and QQ–antibiotic combinations, but also to provide a clearer framework for future research by indicating where the current evidence remains insufficient. In particular, it underscores the need for further well-designed studies to validate the reproducibility of the reported anti-virulence and anti-biofilm effects, to distinguish true QQ activity from broader antimicrobial or indirect biofilm-disrupting mechanisms, and to assess whether these approaches retain efficacy in complex and clinically relevant environments. Moreover, future investigations should address their pharmacological and translational feasibility, including compound stability, bioavailability, toxicity, delivery challenges, and potential performance in in vivo and ultimately clinical settings.

### 3.1. Natural QQ Compounds

Studying natural QQ compounds could be beneficial for eradicating virulence factors of *P. aeruginosa* and *S. aureus*. However, the current lack of animal and clinical studies on this topic remains a limitation and underscores the need for further research to evaluate the translational effectiveness of this approach. Since nanostructures and drug platforms may help overcome the disadvantages of natural compounds—such as rapid metabolism and degradation, water insolubility, and low bioavailability—their use should be considered in forthcoming investigations assessing the antibiofilm efficacy of natural compounds. Consequently, increasing attention has been directed toward the use of nanomaterials as QSIs, as they offer multiple advantages, including improved penetration of biofilms and mucus barriers, enhanced delivery efficiency, greater physicochemical stability, increased solubility, improved biocompatibility, and reduced host toxicity [54,55,56]. Additionally, most existing studies focus on single bacterial species, not taking into consideration that the most challenging pathogens to combat tend to form polymicrobial biofilms [51,57].

Table 3 [49,50,51,52,58,59,60,61] summarizes a few of the recent studies regarding the influence of natural QQ compounds on the *P. aeruginosa* model, while Table 4 [51,62,63,64,65] focuses on the model of *S. aureus*. We took into consideration the outcome of the study, potential complications and limits for medicinal use.

**Table 3:** Examples of studied natural QQ compounds on the *P. aeruginosa* model.

Compound Name	Study Type	Experimental Conditions	Outcome	Limits	Publication Year	Reference
Sesamol	*In vitro*	*P. aeruginosa* PAO1; Sesamol (50–200 μg/mL) tested at sub-MIC concentrations; evaluated alone and in combination with colistin (2 μg/mL).	↓ production of QS–regulated virulence factors, including protease, elastase, pyocyanin, rhamnolipids, and chemotactic activity; ↑ susceptibility of both planktonic and biofilm-associated *P. aeruginosa* cells to colistin.	The best results were accomplished by combining with colistin. Only one strain tested. There were not any in vivo experiments in this study.	2025	[50]
Thymoquinone	*In vitro*	*P. aeruginosa* PAO1; lettuce biofilm model; thymoquinone tested at 0.5–2 mg/mL; evaluated in combination with nisin (1 mg/mL).	↓ QS activity, ↓ expression of virulence-, efflux pump-, and antioxidation-related genes; altered membrane components, ↑ membrane permeability, ↑ susceptibility of biofilms to nisin.	The best results were accomplished by combining with nisin. It does not take into consideration medical use of QQ. There were not any in vivo experiments in this study.	2023	[52]
α-Terpineol	*In vitro* and *In vivo*	*P. aeruginosa* PAO1; C. elegans infection model; Assays included biofilm formation and virulence factor production; MIC for α-Terpineol = 2.4 mg/mL; evaluated both alone and with gentamicin (MIC = 4.0 μg/mL)	↑ QQ activity; ↓ AHL production; ↓ QS- and virulence-related gene expression; ↓ motility; ↓ QS-regulated virulence factors; ↓ biofilm formation; eradication of preformed biofilms; ↑ *C. elegans* survival (73% at 96 h with combination treatment).	The best results were accomplished by combining with gentamycin	2023	[49]
Limonene	*In vitro*	*P. aeruginosa* ATCC 10145; Biofilm model under shear stress conditions. Biofilm grown on different abiotic surfaces (polypropylene, polycarbonate, steel). Tested as a single compound (no combination therapy). Concentration-dependent effect. Concentrations of limonene were ranging from 5 to 40 mL/L.	↓ biofilm on polypropylene from 10^9^ CFU/cm^2^ to 10^4^ CFU/cm^2^, on polycarbonate from 10^9^ CFU/cm^2^ to 10^5^ CFU/cm^2^ and on steel from 10^6^ CFU/cm^2^ to 10^4^ CFU/cm^2^	There was great dependence on the surface where biofilm was studied. There were not any in vivo experiments in this study.	2022	[51]
Geraniol	*In vitro*	*P. aeruginosa* PAO1; study on planktonic cells and biofilm. Evaluated as monotherapy (no antibiotic combination). (0, 0.28, 0.55, 1.1, and 2.2 mg/mL) of geraniol for pyocyanin and elastase activity assay, biofilm assay; 0 and 1.1 mg/mL geraniol for PQS production assay.	↓ planktonic *P. aeruginosa* PAO1 biofilm formation, 0.55–2.2 mg/mL geraniol ↓ elastase production (but 0.28 mg/mL geraniol ↑ elastase production), ↓ PQS production ability, ↓ pyocyanin yield with ↑ geraniol concentration.	Geraniol prolongs the lag phase, and delays the growth period, but with the main population of cells not being inhibited. Low doses (0.28 mg/mL) ↑ elastase production. There were not any in vivo experiments in this study.	2023	[58]
Piperine	*In vitro*	*P. aeruginosa* MTCC 424. Analysis of *lasI* gene expression and biofilm disruption (including preformed biofilm). Tested at sub-inhibitory concentrations (8 and 16 µg/mL) of piperine. Used alone (no combination).	↓ formation of biofilm and ↓ expression of the QS gene (*lasI*) also the ability to disintegrate the pre-existing biofilm of *P. aeruginosa*.	Low bioavailability. There were not any in vivo experiments in this study.	2022	[59]
Fenchone	*In vitro*	MDR-*P. aeruginosa*; Biofilm formation assessed with use of polystyrene flat bottom microtiter tissue culture, results measured at OD595 using an ELISA reader. Tested alone (no antibiotic synergy reported). 0, 0.25, 0.5 and 1 mg/mL fenchone.	Fenchone at 1.0 mg/mL ↓ biofilm production of MDR-PA by 59.15%.	There were not any in vivo experiments in this study. Additional electron microscopic and molecular research is still required to completely understand the mode of action of fenchone against bacteria and candida in order to justify the real-world applications of fenchone as a natural antimicrobial agent.	2022	[60]
2 sunflower oils (AGSUN 5102 CLP and AGSUN 5106 CLP)	*In silico* (molecular docking) and *in vitro.*	*P. aeruginosa* ATCC 27853. Conducted experiments: Inhibition of Cell Attachment, Swarming and Swimming Motility Assays, Pyocyanin Assay. Concentrations of oils: 91.8–11.48 mg/mL (MIC to 1/8 MIC). Compared with azithromycin and cinnamaldehyde.	At MIC, AGSUN 5102 CLP and AGSUN 5106 CLP had 56.64% and 50.34% inhibitory effect on cell attachment, AGSUN 5102 CLP and AGSUN 5106 CLP at MIC showed ↓ of biofilm formation by 62% and 59%.	↑ biofilm formation (−14.52%) was observed after treatment with AGSUN 5106 CLP at 1/8 MIC. Compared to azithromycin and cinnamaldehyde the QQ effect of sunflower oils was weaker. There were not any in vivo experiments in this study.	2023	[61]

Abbreviations: QS—quorum sensing; QQ—quorum quenching; MDR-PA—multi drug resistant *P. aeruginosa*; MIC—minimal inhibitory concentration; *P. aeruginosa*—*Pseudomonas aeruginosa*; *C. elegans*—*Caenorhabditis elegans*; AHL—N-Acyl homoserine lactone; PQS—*Pseudomonas* quinolone signal.

Natural QQ compounds investigated in studies [49,50,51,52,58,59,60,61] show several key advantages, including effective suppression of QS-regulated virulence, strong antibiofilm activity, and significant synergistic interactions with conventional antimicrobials. Most studies are limited to in vitro systems, with insufficient validation under relevant conditions. This applies to sesamol [50], limonene [51], thymoquinone [52], geraniol [58], piperine [59], fenchone [60], and sunflower oils [61]. Only α-terpineol [49] included an in vivo model (*C. elegans*), which, although valuable, does not imitate mammalian infection complexity. The activity of several compounds is strongly influenced by environmental and methodological factors. This is most clearly demonstrated for limonene [51], whose antibiofilm efficacy varied significantly across different surfaces (polypropylene, polycarbonate, steel). A similar limitation applies to thymoquinone [52], which was tested in a lettuce biofilm model, reducing direct clinical relevance. Several compounds exhibit limited standalone activity and achieve optimal effects only in combination with antimicrobial agents. This applies to α-terpineol [49] (gentamicin), sesamol [50] (colistin), and thymoquinone [52] (nisin). Additionally, sunflower oils [61] demonstrated weaker QQ activity compared to reference compounds such as azithromycin and cinnamaldehyde. These findings indicate that many natural QQ agents function more effectively as antibiotic adjuvants rather than conventional therapeutics. Many studies were conducted using a single laboratory strain (primarily PAO1), including α-terpineol [49], sesamol [50], thymoquinone [52], and geraniol [58], which limits extrapolation to clinical settings. Only fenchone [60] included MDR strains, representing a more clinically relevant approach. Across studies [49,50,51,52,58,59,60,61], there is substantial heterogeneity in experimental design, including differences in concentration ranges, assay systems, and biofilm models. This lack of standardization limits cross-study comparability and impedes the development of clinically relevant dosing frameworks. Future research should therefore prioritize validation in mammalian in vivo models, evaluation across diverse clinical and multidrug-resistant isolates, more detailed mechanistic investigation of QS interference, and the standardization of biofilm and dosing models. 

**Table 4:** Examples of studied natural compounds of QQ on the *Staphylococcus aureus* model.

Compound Name	Study Type	Experimental Conditions	Outcome	Limits	Publication Year	Reference
Coumarin	*In silico* (molecular docking), and *in vitro*	MRSA and VRSA; Interaction with virulence-associated proteins using. Compared to simvastatin, and ibuprofen. Tested at half MIC value concentrations (0.763 to 390.6 μg/mL for coumarin).	Coumarin exhibited lower affinity to all virulence factors compared to ibuprofen and simvastatin.	There were not any in vivo experiments in this study; absence of toxicity assessment and clinical strain validation.	2024	[62]
Rhodionin	*In vitro* and mice pneumonia model	MRSA; Study targeting SrtA. Assays: adhesion to fibrinogen, surface protein expression, biofilm formation. Tested alone.	↓ adhesion of *S. aureus* to fibrinogen, ↓ SpA surface expression, ↓ biofilm formation; ↓ SrtA activity (22.85 μg/mL).	More data needed	2022	[63]
Hibifolin	*In vitro* and *in vivo* mice model	MRSA; Enzymatic inhibition of SrtA. Activity evaluated at different concentrations (0 to 256 μg/mL). Synergy with cefotaxime tested. Effect dependent on protein binding sites.	Hibifolin ↓ the virulence of *S. aureus* by inhibiting SrtA activity at IC50 of 31.20 mg/mL	The inhibition activity is ↓ if key binding sites (TRP-194, ALA-104, THR-180, ARG-197, ASN-114) on the SrtA protein are mutated.	2022	[64]
Plantamajoside	*In vitro* and *in vivo* in mice and mammalian cell lines	Tested on the *S. aureus* USA300 both alone and in combination with vancomycin. No QQ activity alone; significant synergistic effect with antibiotic.	Synergy with vancomycin gave the best QQ results. Inhibitory effect on the SrtA at 22.93 µg/mL (35.76 µM).	By itself plantamajoside does not possess any QQ properties.	2025	[65]
Limonene	*In vitro*	Biofilm model of the *S. aureus* ATCC 6538 under shear stress conditions. Biofilm grown on different abiotic surfaces (polypropylene, polycarbonate, steel). Tested as a single compound (no combination therapy). Concentration-dependent effect. Concentrations of limonene were ranging from 5 to 40 mL/L.	The results of biofilm ↓ are surface dependent (polypropylene, polycarbonate and steel were studied). Biofilm ↓ on polypropylene from 106 CFU/cm^2^ to 104 CFU/cm^2^, on polycarbonate from 107 CFU/cm^2^ to 104 CFU/cm^2^ and on steel from 104 CFU/cm^2^ to 103 CFU/cm^2^.	There was great dependence on the surface where biofilm was studied. There were not any in vivo experiments in this study. The greatest reduction was on polycarbonate coupon.	2022	[51]

Abbreviations: QQ—quorum quenching; SpA—*Staphylococcus aureus* protein A; *S.aureus*—*Staphylococcus aureus*; SrtA—Sortase A; MIC—minimal inhibitory concentration; IC50—half-maximal inhibitory concentration; CFU—colony-forming units; MRSA—Methicillin-resistant *Staphylococcus aureus*; VRSA—Vancomycin-resistant *Staphylococcus aureus*.

Natural QQ compounds tested against *Staphylococcus aureus* also show several important limitations. A major weakness is the limited in vivo validation, which applies to coumarin [62] and limonene [51], whereas rhodionin [63], hibifolin [64], and plantamajoside [65] were supported by murine models. Another common issue is incomplete translational characterization, including the lack of toxicity assessment and insufficient validation in broader panels of clinical strains, which was explicitly noted for coumarin [62]. Some compounds also showed restricted standalone efficacy: plantamajoside [65] did not demonstrate meaningful QQ activity alone and performed best only in combination with vancomycin. In addition, surface dependency limited the interpretation of limonene [51], whose antibiofilm efficacy varied markedly across polypropylene, polycarbonate, and steel. Despite these limitations, the studied compounds show several strengths. First, many of them target virulence-related pathways rather than bacterial viability, which is a key advantage of QQ-based strategies. This is particularly evident for rhodionin [63], hibifolin [64], and plantamajoside [65], all of which targets SrtA, resulting in reduced adhesion, diminished surface presentation of virulence proteins, and impaired biofilm formation. Another major advantage is the availability of in vivo support for several compounds, especially rhodionin [63], hibifolin [64], and plantamajoside [65], which makes them more translationally relevant than completely in vitro candidates. Some compounds also showed synergistic potential with antibiotics, most clearly hibifolin [64] with cefotaxime and plantamajoside [65] with vancomycin. Finally, limonene [51] demonstrated measurable antibiofilm activity across several abiotic surfaces, supporting its possible use in anti-biofilm applications. Future studies should focus on in vivo validation, especially for coumarin [62] and limonene [51], as well as on testing in diverse clinical and MDR *S. aureus* isolates. More detailed toxicity and pharmacokinetic studies are also needed, particularly for coumarin [62]. For SrtA-targeting compounds such as rhodionin [63], hibifolin [64], and plantamajoside [65], future work should confirm long-term efficacy in infection-relevant mammalian models and better define their place in combination therapy. In the case of limonene [51], more clinically relevant biofilm models and standardized surface-testing conditions are needed to determine whether its effect can be translated into medical or biomaterial applications.

### 3.2. Synthetic QQ Compounds

QS inhibition represents a promising strategy for controlling bacterial virulence, particularly in pathogens such as *Pseudomonas aeruginosa*. While QSIs production naturally occurs in a wide range of organisms, their practical application is often limited by low production levels and, in some cases, associated toxicity [21]. These limitations have driven increasing interest in the development of synthetic QQ compounds as alternative therapeutic agents.

Among the explored approaches, the design of molecules structurally similar to native AHLs has shown considerable potential. For instance, compounds such as N-(4-{4-fluoroanilino}butanoyl)-L-homoserine lactone (FABHL) and N-(4-{4-chloroanilino}butanoyl)-L-homoserine lactone (CABHL) are similar to HSLs and can interfere with QS systems by targeting key regulatory proteins like LasR [66]. Many researchers have studied molecules that are analogues of the QS systems in *P. aeruginosa*, demonstrating their ability to modulate and inhibit QS, highlighting their potential as anti-virulence agents [67,68,69,70,71].

The development of such synthetic inhibitors is increasingly supported by computational approaches. Molecular docking, for example, enables the analysis of interactions between candidate compounds and their target receptors at the atomic level, providing insights into binding modes and affinities [62]. More broadly, in silico techniques or computer-aided drug design (CADD)—including molecular dynamics simulations, pharmacophores, homology or molecular modelling—play a critical role in identifying and optimizing novel QQ compounds. These methods significantly reduce costs, accelerate the drug discovery process, and minimize reliance on experimental models, making them invaluable tools in the search for effective QS inhibitors [48,72].

In analogy to the natural compounds described in the previous section, selected recent studies investigating synthetic quorum quenching (QQ) compounds are summarized in Table 5 [73,74,75,76,77,78,79] and Table 6 [80,81,82,83].

**Table 5:** Effects of selected synthetic QQ compounds on the *Pseudomonas aeruginosa* models.

Compound Name	Study Type	Experimental Conditions	Outcome	Limits	Publication Year	Reference
Fifteen novel benzo[d]thiazole/quinoline-2-thiol bearing 2-oxo-2-substitutedphenylethan-1-yl compounds	*In silico* and *in vitro*	*Pseudomonas aeruginosa* ATCC 27853; Primarily in silico (molecular docking) + limited in vitro validation. Biofilm and antimicrobial assays at 50 μg/mL, 100 μg/mL, 150 μg/mL, 200 μg/mL and 250 μg/mL. Tested as single agents (no synergy reported).	3/15 compounds (3-R1 = benzo[d]thiazol-2-yl, R2 = 2,3-dihydrobenzo[b][1,4]dioxin-6-yl; 6-R1 = benzo[d]thiazol-2-yl, R2 = 3-methoxyphenyl; 7-R1 = benzo[d]thiazol-2-yl, R2 = 2,4-dimethoxyphenyl) exhibited promising QSI (IC50 of 115.2 μg mL^−1^ and 182.2 μg mL^−1^, and 45.5 μg mL^−1^, respectively) and selectivity inhibit the *LasB* over the PqsR QS system. 1/15 (7-R1 = benzo[*d*]thiazol-2-yl, R2 = 2,4-dimethoxyphenyl) compound showed clearly anti-biofilm formation with the ability to ↓ of 70% biofilm biomass at 250 μg/mL.	Moderate cytotoxicity; Docking only; No experimental validation; Low solubility (limited bioavailability). There were not any in vivo experiments in this study.	2021	[73]
Squalenyl hydrogen sulfate nanoparticle (SqNP)	*In vitro* and *in vivo*	*P. aeruginosa* PA14 Study targeting PqsR system. Assessed PQS, HHQ, pyocyanin and eDNA secretion. Dose-dependent analysis (SqNP constantly 20 × 10^−6^ M). Tested also with tobramycin (between 6.25 μg/ mL^−1^ to 200 μg/ mL^−1^	SqNP effectively inhibits the PqsR QS system, ↓ Pyocyanin production at IC_50_ value 200 × 10^−9^ M, ↓ *PqsR* reporter-gene at IC_50_ 11 × 10^−9^ M, ↓ Biofilm components like eDNA.	Possible Interaction with CNS transporters (dopamine, norepinephrine), Some analogues inhibited CYP enzymes (possible interactions with other drugs).	2021	[79]
Cobalt (II, III) oxide (Co_3_O_4_) nanoparticles	*In vitro*	Clinical isolates of *P. aeruginosa*; Tested at multiple concentrations (tested over a concentration range obtained by two-fold serial dilutions, starting from 10 mg/mL (10,000 µg/mL). The final concentrations included 10,000, 5000, 2500, 1250, 625, 312.5, and 156.25 µg/mL). Monotherapy.	Biofilm was inhibited to 76.54% at 10 mg/mL and 0.156 mg/mL	*P. aeruginosa* biofilms and persister cells can benefit from applying co3o4-NPs. There were not any in vivo experiments in this study.	2023	[74]
3-Hydroxypyridin-4(1*H*)-one Derivatives	*In vitro* and *in vivo C. elegans* model	*P. aeruginosa* PAO1. Enzymatic/QS inhibition assays. IC50 determined. Synergy with ciprofloxacin (MIC 0.25 μg/mL) and tobramycin (MIC 5.00 μg/mL) tested.	The most potent derivative 16e was identified as a selective inhibitor of the *pqs* system (IC50 = 3.7 μM) and its related virulence factor pyocyanin (IC50 = 2.7 μM).	Some of the 3-hydroxypyridin-4-one derivatives have demonstrated toxicity at higher doses, harder to define a therapeutic window.	2023	[75]
c-di-GMP G-quadruplex inducer hybrids	*In vitro* and *in vivo C. elegans* model	*P. aeruginosa* PAO1 and its hybrids. Mechanism independent of classical QS receptors. Tested in synergy with tetracycline (2.5 μg). Concentrations tested: 0.625, 1.25, 2.5, 5, and 10 μM.	Hybrids A7 (C20H17N2) and A11 (C20H16FN2) attenuated virulence factors and ↓ the motility phenotypes of *P. aeruginosa.*	Does not act on regulatory systems but acts directly on the signalling molecule (c-di-GMP).	2024	[76]
Fourteen benzo[d]thiazoles	*In silico* and *in vitro*	*P. aeruginosa* PAO1; Selected compounds further tested in combination with antibiotics and antifungal drug (imipenem, azithromycin, fluconazol) (synergistic conditions). Anti-virulence assays were conducted at sub-MIC levels to exclude growth-inhibitory effects. Although sub-MIC values differed among compounds, the most potent derivatives (3c, 3e, and 8d), which demonstrated the highest inhibition of biofilm formation, were evaluated at a concentration of 9.375 µg/mL.	3/14 compounds (2-(Benzo[*d*]thiazol-2-yl)-3-(4-(4-methylpiperazin-1-yl)phenyl)acrylonitrile, 2-(Benzo[*d*]thiazol-2-yl)-3-(4-(pyrrolidin-1-yl)phenyl)acrylonitrile, 2-(7-(4-Fluorophenyl)pyrazolo [1,5-a]pyrimidin-3-yl)benzo[*d*]thiazole) exhibited the highest percentage inhibition in biofilm formation (77%, 63.9%, 69.4%), pyocyanin production (74.6%, 64.9, 69.4%), and rhamnolipids production (58.5%, 51%, 54.3%) in *P.aeruginosa*. 3c dissociates Las R inhibitory IC50 = 1.37 ± 0.35 μM.	Only 3 from 14 new synthesized compounds showed effect, synergy with antibiotics, lack of toxicity testing. There were not any in vivo experiments in this study.	2024	[77]
2-(4-bromophenyl)-N-(2-oxotetrapyridinefuran-3-yl	*In silico* (molecular docking), *in vitro* and *in vivo C. elegans*	*P. aeruginosa* PAO1; inhibitory effects of compound no.3 and compound no.10 on biofilm formation were identified at different concentrations ranging from 50 to 400 μM. Also: motility assay, pyocyanin production, elastase analysis and QS-regulated genes expression.	Compound no.10: ↓ pyocyanin production by 65.29%. ↓ more than 60% of biofilm formation at above 200 μM. ↓ the motility zones, ↓ the expression of *lasI*, *lasR*, *rhlI*, *rhlR*, and *mvfR*.	Tests should also be repeated on clinical drug-resistant strains and assessment of the therapeutic effect of compound no.10 on the infected animal model.	2022	[78]

Abbreviations: QQ—quorum quenching; *P. aeruginosa*—*Pseudomonas aeruginosa*; QSI—quorum sensing inhibitor; Co_3_O_4_—cobalt oxide; c-di-GMP—cyclic di-guanosine monophosphate; IC50—half-maximal inhibitory concentration; SqNP—Qualenyl hydrogen sulfate nanoparticle; TACN—1,4,7-triazacyclononane; CYP—Cytochromes P450; CNS- central nervous system; PQS—pseudomonas quinolone signal; HHQ—4-hydroxy-2-heptylquinolinek; QS—quorum sensing; *C. elegans*—*Caenorhabditis elegans*; HHQ—2-heptyl-4-quinolone; MIC—minimal inhibitory concentration.

Synthetic QQ compounds targeting *P. aeruginosa* show several limitations. A major issue is limited in vivo validation, which applies to benzo[d]thiazole/quinoline derivatives [73], Co_3_O_4_ nanoparticles [74], and benzo[d]thiazoles [77], while only selected compounds such as SqNP [79], 3-hydroxypyridin-4-one derivatives [75], and c-di-GMP hybrids [76] were tested in *C. elegans* or partial in vivo systems. Moderate cytotoxicity and pharmacological risks were reported for benzo[d]thiazole/quinoline derivatives [73], while SqNP [79] showed potential interactions with CNS transporters and CYP enzymes, leading to concerns about possible off-target interactions. Several studies also relied on single reference strains (PAO1 or ATCC 27853), including [73,75,76,77,78], limiting clinical relevance. Additionally, heterogeneity of experimental conditions and concentration ranges across studies which complicates comparison and dose translation. Finally, some compounds exhibited toxicity at higher concentrations (3-hydroxypyridin-4-one derivatives [75] or lacked comprehensive toxicity evaluation [77]. Despite these limitations, synthetic QQ compounds offer several key advantages. To begin with, many demonstrate high potency and target specificity, particularly toward defined QS systems such as PqsR, as seen for SqNP [79] and 3-hydroxypyridin-4-one derivative 16e [75] (low IC_50_ values). Furthermore, several compounds exhibit strong antibiofilm and antivirulence activity, including benzo[d]thiazoles [77], which significantly reduced biofilm, pyocyanin, and rhamnolipid production, and compound no.10 [78], which downregulated multiple QS genes (*lasI*, *lasR*, *rhlI*, *rhlR*, *mvfR*). Finally, synergistic effects with antibiotics were observed for multiple compounds, including 3-hydroxypyridin-4-one derivatives [75], c-di-GMP hybrids [76], and benzo[d]thiazoles [77], highlighting their potential as adjuvants.

Importantly, some compounds introduce novel mechanisms beyond classical QS inhibition, such as c-di-GMP G-quadruplex hybrids [76], which act directly on intracellular signalling molecules rather than receptor pathways. Moreover, the flexibility of synthetic design allows for structural optimization and improved selectivity, offering a significant advantage over natural compounds. Future research should focus on comprehensive in vivo validation in mammalian models, especially for [73,74,77], to confirm efficacy and safety. Studies should also expand to clinical and MDR isolates, addressing the current reliance on reference strains [73,77]. Given the promising results, systematic evaluation of combination therapy with antibiotics should be prioritized [75,76,77].

**Table 6:** Effects of selected synthetic QQ compounds on the *Staphylococcus aureus* model.

Compound Name	Study Type	Experimental Conditions	Outcome	Limits	Publication Year	Reference
ASA and trifluoperazine	*In silico* (molecular docking) and *in vitro* (only antimicrobial part).	*S. aureus* (ATCC 29213); Tested both alone and comparatively. Concentrations tested: 1000, 750, 500, 250, and 200 µg/mL.	Biofilm inhibition activity at 250 µg/mL. In contrast to ASA, trifluoperazine showed no antimicrobial activity at any tested concentration.	Repurposing drugs might be problematic from a legal point of view (trifluoperazine is antipsychotic drug), only docking showed effect on the QS, the convergence of laboratory experiments and computational predictions was not entirely consistent. There were not any in vivo experiments in this study.	2024	[80]
1-Methyl-2-phenylpyridin-1-ium Derivatives	*In vitro* and *in vivo* mice model.	*S. aureus* ATCC 25923; Assays targeting FtsZ and membrane integrity. Single infection model. Monotherapy.	The compounds inhibit bacterial division by targeting FtsZ, increasing membrane permeability and disrupting proton gradients.	Only one infection type was evaluated. Without full pharmacokinetic profiling, it is difficult to evaluate clinical feasibility or dosing strategies.	2025	[81]
Chitosan quaternary ammonium salts: DMDAC, DINCC, DMDBC	*In vitro* and *in vivo* on L929 cells.	Monotherapy	Chitosan derivatives eliminated more than 90% of the mature *S. aureus* biofilm when applied at a concentration of 2.5 mg/mL.	The antibacterial properties are related to the hydrophobicity of substituents.	2024	[82]
Tetrahydropyridine Analogues	*In silico* (molecular docking) and *in vivo.*	*S. aureus*, 3 compounds tested with different concentrations 100^−5^ μg/mL.	The highest level of biofilm eradication (75.08 ± 0.84%) was achieved by compound 4n, with compound 4l following at 69.69 ± 4.23%. The lowest eradication effect was observed for compound 4k, at 68.85 ± 3.75%.	Compounds were tested on singular strain.	2026	[83]

Abbreviations: MIC—Minimum Inhibitory Concentration, QS—quorum sensing; *E. coli*—*Escherichia coli*; *S. aureus*—*Staphylococcus aureus*; *E. faecalis*—*Enterococcus faecalis*; ASA—acetylsalicylic acid; FtsZ—protein, Filamenting temperature-sensitive mutant Z″; DMDAC—N′,N′-dimethyl-N′-dodecyl-ammonium chloride-N-amino-acetyl chitosan; DINCC—N′-dodecyl-N-isonicotinyl chitosan chloride; DMDBC—N′,N′-dimethyl-N′-dodecyl-ammonium chloride-N-benzoyl chitosan.

Limited in vivo evidence remains an issue for ASA and trifluoperazine [80], whereas 1-Methyl-2-phenylpyridin-1-ium derivatives [81], chitosan quaternary ammonium salts [82], and tetrahydropyridine analogues [83] include in vivo components yet still lack comprehensive translational validation. Another constraint is restricted experimental scope, such as single infection models in 1-methyl-2-phenylpyridin-1-ium derivatives [81] and testing on single laboratory strains in [83]. Additionally, pharmacological and regulatory considerations present challenges for trifluoperazine [80] due to its primary use as an antipsychotic, complicating application to alternative clinical use. Finally, [81] is characterized by a lack of pharmacokinetic data and clearly established dosing regimens, whereas mechanistic uncertainty regarding antibiofilm activity persists for materials such as chitosan derivatives [82], whose effects are strongly influenced by physicochemical properties, including hydrophobicity. These compounds demonstrate several important strengths. Many show strong antibiofilm activity, particularly chitosan derivatives [82], which removed >90% of mature biofilm, and tetrahydropyridine analogues [83], achieving ~70–75% eradication. Several compounds act via novel or non-classical mechanisms, such as FtsZ inhibition and membrane disruption by 1-methyl-2-phenylpyridin-1-ium derivatives [81], which may indirectly affect QS-regulated phenotypes. Future studies should prioritize robust in vivo validation and expanded infection models, especially for ASA/trifluoperazine [80]. Comprehensive pharmacokinetic and toxicity profiling is essential, particularly for systemically active compounds. 

Across the analyzed studies, both natural (e.g., terpenes, phenolics, plant-derived metabolites) and synthetic or semi-synthetic compounds demonstrate consistent ability to interfere with key QS-associated processes, including AIs production and downstream regulation of virulence determinants. In *P. aeruginosa*, many compounds effectively modulate the Las, Rhl, and Pqs systems or associated signalling networks, leading to reduced production of pyocyanin, elastase, rhamnolipids, and extracellular DNA, as well as impaired motility and biofilm formation. In *S. aureus*, a slightly different but functionally analogous strategy is observed, with compounds targeting SrtA, or membrane-associated processes, ultimately limiting adhesion, surface protein anchoring, and biofilm maturation. These findings highlight that, despite differences in molecular targets between Gram-negative and Gram-positive bacteria, QQ-based approaches converge on a shared functional outcome: attenuation of virulence and disruption of biofilm-associated persistence. 

### 3.3. Application of Nanoparticles (NPs) in Quorum Quenching

Recent progress in NP research has underscored their potential as antimicrobial and antipathogenic agents, particularly due to their ability to interfere with biofilm formation and QS systems. However, despite these promising attributes, their application in clinical settings remains constrained by several biological, toxicological, and translational challenges that require careful consideration [84].

NPs, typically ranging from 1 to 100 nm in size, possess unique physicochemical properties, including a high surface area-to-volume ratio, tunable surface chemistry, and enhanced reactivity. These features have led to their extensive investigation as delivery platforms for a wide range of therapeutic agents, including antibiotics and anticancer drugs [85,86]. In the context of QQ, NPs may function both as carriers of QSIs and as active agents capable of modulating bacterial communication pathways.

NPs can exert intrinsic antimicrobial and potential anti-QS effects through multiple mechanisms. One of the primary modes of action involves the release of metal ions from the NP surface, which can induce toxicity in bacterial cells. Additionally, NPs may promote the generation of ROS, leading to oxidative stress and damage to cellular components, including proteins, lipids, and nucleic acids. Direct interaction with bacterial membranes may potentially result in structural disruption, membrane depolarization, and increased permeability due to alterations in surface charge and integrity [32]. These processes may indirectly affect QS-regulated phenotypes, including virulence factor production and biofilm formation, although their effects are often difficult to distinguish from general antimicrobial stress. However, such nonspecific mechanisms raise concerns regarding off-target effects, including cytotoxicity toward host cells and disruption of commensal microbiota [87,88,89,90,91]. 

NPs have been increasingly investigated as potential delivery systems for QSIs derived from both natural and synthetic sources. Their tunable properties enable targeted delivery, controlled release, and improved stability of bioactive compounds, potentially enhancing therapeutic efficacy. However, their clinical application remains highly limited due to persistent challenges related to safety, biodistribution, standardization, and translational validation [92].

Furthermore, incorporation of NPs into biomedical materials, such as wound dressings, gels, and ointments, allows localized and sustained delivery of antimicrobial activity at infection sites. This approach is particularly relevant in chronic wounds and burn injuries, where biofilm-forming pathogens significantly impair healing. Nevertheless, the effectiveness of NP-based delivery systems is strongly influenced by physicochemical parameters such as particle size, surface charge, coating, and aggregation behaviour, which remain insufficiently standardized across studies [32,85].

Metal-based NPs, including silver, zinc oxide (ZnO), and titanium dioxide (TiO_2_), have been extensively investigated for their QQ and antibiofilm activities [32,93,94,95,96,97]. Zn^2+^ ions and ZnO NPs have been shown to exhibit antivirulence effects against *P. aeruginosa* at sub-inhibitory concentrations (<3 mM). These NPs can reduce the production of key virulence factors, including elastase, pyocyanin, and biofilm components, likely through ROS generation, membrane disruption, and intracellular accumulation [21,96]. However, their dose-dependent cytotoxicity and potential adverse effects on host tissues remain important limitations. Similarly, titanium dioxide NPs (TiO_2_NPs) have demonstrated the ability to interfere with QS systems and antibiotic resistance mechanisms in multidrug-resistant *P. aeruginosa*. Experimental studies indicate that TiO_2_NPs can significantly reduce biofilm formation and downregulate the expression of QS-related genes (*lasR*, *lasI*, *rhlI*, *rhlR*, *pqsA*, *pqsR*) as well as efflux pump genes (*mexA*, *mexB*, *mexY*), thereby enhancing susceptibility to conventional antibiotics [98].

Despite extensive in vitro evidence supporting the efficacy of NPs in QS inhibition, their translation into clinical practice remains limited. A key challenge is the discrepancy between in vitro and in vivo conditions, as most studies rely on simplified laboratory models that do not accurately reflect the complexity of host environments. Moreover, current research predominantly focuses on reference strains such as *P. aeruginosa* PAO1 and PA14. This represents a major limitation, as the phenotypic traits, virulence profiles, and responses to antivirulence interventions observed in PAO1 and PA14 may not adequately reflect those of clinical isolates, particularly mucoid CF strains, MDR/XDR variants, and biofilm-associated isolates from chronic wound infections [21,32].

Another major concern is the potential toxicity and long-term accumulation of NPs in host tissues. The high surface-to-volume ratio can enhance interactions with different biological components when these particles are inhaled, absorbed, or ingested. Furthermore, their capacity to penetrate cellular barriers and their resistance to biodegradation contribute to an increased toxic potential of these nano-entities [99,100]. Toxicity assessments should take into account the mechanisms underlying both acute and chronic effects, the breakdown of particulate materials, any resulting activation of defensive or inflammatory responses, metabolic processes, long-term toxicity, and the pathways of elimination in both cellular and animal models [99].

In addition, their interaction with host microbiota may lead to unintended dysbiosis, further complicating their clinical application. Importantly, NP exposure may also impose selective pressure on microbial populations [87,91].

Environmental considerations also represent a critical aspect of NP application. The increasing use of NPs may lead to their accumulation in natural ecosystems, where they can affect microbial communities and disrupt ecological balance, raising concerns about long-term environmental safety [88,89,90].

Although NP-based QQ strategies hold considerable promise, their clinical implementation requires a more comprehensive understanding of their mechanisms of action, safety profiles, and long-term effects. Standardization of NP synthesis, characterization, and biological evaluation is essential to ensure reproducibility and comparability across studies [101,102]. Furthermore, well-designed in vivo studies and appropriate animal models are necessary to assess pharmacokinetics, biodistribution, efficacy, and toxicity under physiologically relevant conditions [74,84,103].

In summary, while NPs represent a promising platform for both direct QQ and the delivery of QS inhibitors, their application is currently limited by significant toxicological, environmental, and translational challenges. Addressing these limitations will be essential before NP-based QQ strategies can be considered viable alternatives for the treatment of bacterial infections, particularly those caused by drug-resistant pathogens.

## 4. Experimental and Disease Models of QQ in Medicine

### 4.1. Lung Infections (Cystic Fibrosis Model)

CF is an autosomal recessive disorder resulting from mutations in the cystic fibrosis transmembrane conductance regulator (CFTR) gene. The CFTR protein functions as an epithelial anion channel responsible for chloride and bicarbonate transport across the membranes of respiratory epithelial cells, thereby playing a central role in maintaining airway homeostasis. Under physiological conditions, proper CFTR activity preserves hydration of the epithelial surface, helps prevent bacterial persistence, and supports correct mucin unfolding in the lungs. By contrast, mutations in the CFTR gene can either impair or completely eliminate the function of the channel, resulting in the accumulation of abnormally thick mucus within the respiratory tract [104]. Chronic pulmonary infections, predominantly driven by antibiotic-resistant pathogens adapted to the unique microenvironment of the CF airway, represent the principal cause of morbidity and mortality in people with CF (pwCF) [105,106].

At present, chronic administration of inhaled antibiotics remains the standard therapeutic approach for controlling persistent bacterial infections in patients with CF. In contrast to oral or intravenous administration, aerosolized delivery enables the achievement of high local drug concentrations within the respiratory tract, thereby optimizing pulmonary pharmacokinetic/pharmacodynamic exposure while minimizing systemic toxicity. Nevertheless, therapeutic efficacy is critically determined by the pathophysiological characteristics of the CF lung microenvironment, which substantially modulate antibiotic deposition, diffusion, and antimicrobial activity following inhalation. CF airway secretions exhibit altered rheological and biochemical properties, including increased viscosity, abnormal mucin organization, and a distinct inflammatory and ionic milieu, compared with healthy airways. In parallel, biofilm formation by CF pathogens and the dense mucus matrix act as major diffusion barriers, limiting antimicrobial penetration and contributing to bacterial persistence. A more detailed understanding of these local determinants is therefore essential for the rational optimization of inhaled antimicrobial regimens directed against CF-associated pathogens [106,107].

Among the pathogens implicated in advanced CF lung disease, *P. aeruginosa* remains the most prevalent and clinically significant, with the Cystic Fibrosis Foundation Patient Registry reporting its presence in 28.4–43.2% of adult with pwCF [108]. *P. aeruginosa* is a highly virulent opportunistic pathogen capable of inducing extensive pulmonary damage through the secretion of a broad spectrum of virulence factors [109]. Management of *P. aeruginosa* infection is primarily based on cyclic or continuous inhaled antibiotic therapy, most commonly with aztreonam, tobramycin, and colistin. Nevertheless, the long-term selective pressure exerted by these regimens promotes the emergence of antibiotic-resistant *P. aeruginosa* strains, thereby reducing therapeutic effectiveness and contributing to substantial morbidity and mortality [104]. Biofilm-associated growth is a major determinant of *P. aeruginosa* persistence in the CF airway and is also thought to contribute to the development of pulmonary exacerbations. Limited penetration of antimicrobial agents through the biofilm matrix is considered one of the principal mechanisms responsible for the insufficient efficacy of anti-pseudomonal therapy [107]. Although biofilms are classically described as surface-attached microbial communities forming on damaged tissue or foreign bodies such as urinary catheters, the situation in CF lungs appears distinct. In the lungs of individuals with CF, however, bacteria frequently form self-aggregating clusters suspended within the airway mucus rather than attaching to a solid surface [10,110].

In recent years, multiple experimental studies have evaluated QS inhibitors (QSI) against *P. aeruginosa*, as summarized in Table 3 [49,50,51,52,58,59,60,61]. Most of these studies have focused on natural compounds. Naturally derived agents are of particular interest in medical research because they are generally biodegradable and often supported by a history of prior biological use, making them attractive candidates for the control of bacterial and fungal infections. However, compared with synthetic compounds, they are frequently characterized by lower potency, reduced stability, and less well-defined pharmacokinetic profiles [21].

Importantly, QS in *P. aeruginosa* also mediates interspecies interactions that may influence disease progression in CF. Sass et al. demonstrated that *P. aeruginosa* laboratory isolates can interact synergistically with the antifungal azole voriconazole, resulting in inhibition of biofilm metabolism in several *Aspergillus fumigatus* laboratory strains; this effect was mediated primarily by pyoverdine, but also by pyocyanin and pyochelin [111]. In addition, PQS has been implicated in intermicrobial communication, ferric iron (Fe^3+^) chelation, and iron delivery to the bacterial cell membrane in cooperation with siderophores. Under iron-limited conditions, PQS inhibited *A. fumigatus* biofilm formation, whereas in an iron-rich environment both fungal growth and biofilm formation were significantly enhanced compared with iron-depleted conditions. Notably, this phenotype was not observed in an *Aspergillus* mutant lacking siderophore expression. These findings suggest that PQS production by *P. aeruginosa* may promote fungal persistence in CF airways, where iron homeostasis is frequently dysregulated, thereby potentially aggravating disease severity [112].

Mucus viscosity and oxidative stress in cystic fibrosis lungs are both likely to reduce the effectiveness of QQ against *P. aeruginosa*, although direct CF-specific evidence for many QQ agents remains limited [113]. Garbero et al. showed that in a biomimetic cystic fibrosis mucus model, all tested QS classes (phenazines, lactones and quinolones) molecules were able to cross the mucus barrier, but their diffusion was heterogeneous, ranging from 26% to 64%, with retention governed mainly by chemical interactions with mucins rather than steric filtering alone. Importantly, when the CF mucus layer was integrated with a membrane permeability model, pyocyanin permeability decreased by about 60%, indicating that the pathological mucus environment can selectively limit the transport and bioavailability of bacterial signalling molecules and, by extension, may also hinder the activity of QQ agents [113]. Oxidative stress may further limit the efficacy of QQ approaches in CF by creating a selective environment that favours bacteria with intact or resistant QS-systems. García-Contreras et al. demonstrated that oxidative stress enhances the advantage of strains capable of mounting a robust QS-dependent antioxidant response and, importantly, that exposure to H_2_O_2_ together with the QS inhibitor, furanone C-30, increased the proportion of resistant to furanone C-30 mutants of *P. aeruginosa*, indicating that oxidative stress can promote resistance to QQ [114].

After *P. aeruginosa*, *S. aureus* represents the second most frequently encountered pathogen in CF airways. Additional microorganisms implicated in CF lung infection include *Burkholderia* spp., non-tuberculous mycobacteria (NTM), *Stenotrophomonas maltophilia*, *Achromobacter* spp., and fungi such as *Aspergillus* spp. [10]. To counter resistance and virulence, anti-virulence therapies aim to disarm *S. aureus* without exerting lethal pressure, thereby reducing resistance selection. QSI targeting the agr system disrupt toxin production and biofilm dispersion, though the clinical translation is challenged by paradoxical biofilm reinforcement in chronic infections [115,116].

Therefore, effective anti-virulence strategies based on QQ should consider the complex genetic background of biofilm formation (e.g., QS genes, EPS extracellular matrix genes, related regulatory networks) and may require combined approaches that target both QS regulation and biofilm structural mechanisms.

### 4.2. Chronic Wound Infections, Ulcers and Burns 

Chronic surgical wound infections (CSWIs) represent a significant burden in clinical practice, leading to prolonged hospitalization, increased healthcare costs, and substantial patient morbidity [117]. CSWIs remain a major clinical challenge, as biofilm formation promotes antimicrobial tolerance and impairs wound healing. Current evidence suggests that biofilm-forming microorganisms are present in 60–80% of chronic wounds, although data from low-resource settings remain limited [118]. Common pathogens such as *S. aureus*, *P. aeruginosa*, and *E. coli* frequently form biofilms, further complicating infection management [119].

*P. aeruginosa* forms highly impermeable biofilms that are exceptionally difficult to eradicate even with the broad range of therapeutic options available to clinicians, thereby significantly contributing to the chronicity of these wounds due to the frequent re-establishment of biofilms following debridement [120].

The spectrum of bacteria colonizing burn wounds has evolved over time, largely in response to shifts in antibiotic use and emerging resistance patterns. In patients with severe burns, altered tissue pharmacokinetics may lead to suboptimal local antibiotic concentrations, thereby compromising therapeutic efficacy and favouring the selection of resistant strains. As a result, multidrug-resistant (MDR) organisms, including MRSA, *Enterococcus* spp., *Pseudomonas* spp., and *Acinetobacter* spp., have become increasingly prevalent and represent a major challenge in burn wound management [121]. Gram-positive bacteria are typically associated with early burn wound colonization, whereas Gram-negative organisms tend to predominate at later stages of injury. Among Gram-positive pathogens, *S. aureus* remains the most prevalent globally and is strongly associated with increased morbidity and mortality, particularly in cases of invasive burn wound infection [122].

Antimicrobial wound management may involve several therapeutic modalities, including wound irrigation, gels, and antimicrobial dressings or pads. Their clinical use depends on multiple factors, such as the nature of the active agent, its concentration, antimicrobial spectrum, and cytotoxic potential. Although a range of wound-care products with proven antimicrobial efficacy and relatively low cytotoxicity is currently available, this does not support indiscriminate or prolonged use. Given the limitations of existing agents, particularly restricted exposure time and residual cytotoxicity, there remains a clear need to identify novel and safer therapeutic molecules [123,124,125].

Given the limitations of existing agents, particularly restricted exposure time and residual cytotoxicity, there remains a clear need to identify novel and safer therapeutic molecules [103,104,105], among which QQ represents a promising approach; however, its clinical translation is still constrained by factors such as limited stability, delivery challenges, and variable efficacy in complex infection environments. The chronic wound microenvironment, marked by sustained neutrophil-driven ROS production and elevated metalloproteinase activity, promotes extracellular matrix (ECM) degradation and biofilm formation. As highlighted by studies on chronic wound pathology, this highly inflammatory and protease-rich milieu not only supports microbial persistence but may also limit QQ efficacy—particularly for enzyme-based QQ agents—by promoting their proteolytic degradation and functional inactivation, while simultaneously facilitating the formation of structured, biofilm-based communities that are inherently less susceptible to QS disruption [126].

Nanostructured drug delivery systems may enhance topical antimicrobial therapy in burn wounds by improving local drug availability while reducing systemic exposure. However, their therapeutic performance depends on burn severity, wound infection status, and NP characteristics, whereas optimal formulations should ensure biocompatibility, low toxicity, biodegradability, and controlled drug release [121,127,128].

### 4.3. Implant-Associated Infections (IAIs)

Orthopedic implant surgeries have revolutionized musculoskeletal care by improving functional recovery and quality of life. However, postoperative infections associated with implants remain one of the most serious complications, leading to prolonged hospitalization, increased healthcare costs, and functional impairment. The global incidence of surgical site infections after orthopedic implant surgery ranges between 1% and 10%, depending on surgical type, patient factors, and infection control measures [129].

Microbiological analysis suggests that *S. aureus* persists as the primary etiologic agent of post-operative infections [129], followed by *P. aeruginosa* and *E. coli*. These findings align with those of Alelign et al. [130] who identified Gram-positive cocci as the dominant group in 42% of orthopedic infections. However, the growing detection of Gram-negative pathogens such as *Klebsiella pneumoniae* and *Acinetobacter baumannii* signifies a shift toward nosocomial infections with multidrug-resistant profiles, corroborating findings from Mussab et al. [131], and Elifranji et al. [132].

The pathogenesis of implant-associated infections (IAIs) remains a fundamental challenge when it comes to their effective prevention. Following implantation, the sterile surface is rapidly conditioned by host proteins; while this process is essential for tissue integration, these proteins simultaneously serve as ligands for bacterial adhesins. Although initial bacterial attachment is reversible, it typically transitions into biofilm formation within hours. The subsequent secretion of an EPS stabilizes this structure, providing the biofilm with significant resistance to both host immune defences and systemic antibiotics. As the biofilm matures and localized nutrient sources become depleted, microbial cells begin to disperse, facilitating the colonization of adjacent tissues and the further spread of the infection [133,134].

A significant limitation of current strategies for minimizing IAIs is that they do not directly address the implant surface, which remains the primary site for bacterial colonization and biofilm development. To address this, antimicrobial implant coatings have been engineered to help prevent and treat these infections [135]. These technologies function through distinct mechanisms: passive surface modifications alter the physico-chemical properties of the implant to inhibit bacterial adhesion without agent release, whereas active surface modifications incorporate pharmacologically active compounds, such as silver (0.33–2.89 g silver on the surface of titanium prostheses) or iodine (10–12 μg/cm^2^), to exert direct antimicrobial effects. Despite their theoretical potential, the clinical efficacy of these coatings remains poorly defined. A critical gap in current research is the lack of comparative studies between different coating materials [136]. Existing data are largely fragmented across small, single-centre trials, with very few head-to-head assessments available. Consequently, there is insufficient evidence to provide clear guidance for implant selection, particularly when attempting to balance infection prevention against concerns regarding biocompatibility and cost [134].

Fong et al. [137] demonstrated that disulfide bond-containing ajoene analogues effectively attenuate Pseudomonas aeruginosa infection in a murine IAIs model. The study showed that treatment with these QSI significantly reduced bacterial virulence in vivo, without directly targeting bacterial viability. This effect was linked to the suppression of QS-regulated factors such as elastase, rhamnolipids, and pyocyanin, which are essential for biofilm formation and persistence on implanted materials. These findings highlight QSI as a promising strategy for controlling biofilm-related infections on medical devices [137].

In a similar context, QS-targeted interventions have also been explored in Gram-positive pathogens involved in IAIs. Brady et al. [138] demonstrated that inhibition of QS in *S. aureus*, particularly via the RNAIII-inhibiting peptide (RIP), can reduce biofilm formation on orthopedic implants. This approach interferes with agr signalling, limiting virulence expression and bacterial attachment, which are critical steps in IAIs development [138].

Beyond soluble QS inhibitors, another important direction involves the development of anti-infective biomaterial surfaces with intrinsic QQ properties. Vogel et al. [139] reported the immobilization of the QQ enzyme PvdQ—an N-terminal nucleophile acylase encoded within the pyoverdine (pvd) gene cluster—onto polydimethylsiloxane (PDMS) silicone surfaces. The resulting functionalized material retained QQ activity and significantly limited bacterial colonization on indwelling medical devices, including urinary and intravascular catheters [139].

## 5. Clinical and Translational Risks of Quorum Quenching (QQ)

Despite the promising anti-virulence potential of QQ strategies, their clinical translation remains constrained by several unresolved biological, ecological, and evolutionary uncertainties. In particular, the risks of tolerance development and unintended disruption of host-associated microbiota underscore the need for comprehensive investigation before widespread therapeutic implementation.

### 5.1. Risk of Developing Tolerance and Functional Resistance 

Bacteria have developed a wide range of molecular mechanisms to survive in extreme conditions, from nutrient limitation to environmental factors. Unlike antibiotics, QSIs act by interfering with QS-mediated gene expression controlling virulence rather than bacterial growth and are therefore considered to impose lower selective pressure for bacterial resistance development. However, resistance to QS inhibition may still emerge. The genetic plasticity of microorganisms and their ability to acquire resistance genes through horizontal gene transfer could hinder the long-term effectiveness of QSI [140]. Nonetheless, this area remains relatively understudied, with only limited reports describing potential QQ-related resistance mechanisms [141]. Maeda et al. [142] were among the first to demonstrate experimentally that bacteria can evolve resistance to QQ. Using *P. aeruginosa* as a model organism, the authors tested a widely used QQ compound—the synthetic brominated furanone 4-bromo-5-(bromomethylene)-2(5H)-furanone, known as C-30. In transposon mutants, resistance was associated with mutations in mexR and nalC, which encode repressors of the MexAB–OprM resistance operon [143]. These mutations enhanced efflux of the QQ compound, reducing its ability to inhibit several QS-regulated virulence factors and pathogenicity. Consistent with this, C-30 showed diminished efficacy in mexR mutants. Critically, clinical isolates from CF patients (Liverpool epidemic strain 12142) also carried mexR/nalC mutations, suggesting that even in the absence of the QS inhibitor, cells may naturally evolve resistance to QQ compounds in the pathogenic state when confronted with the pressures of antibiotic treatment, and due to standing genetic variation. Hence, antibiotic treatment may incidentally reduce susceptibility to QQ compounds. However, spontaneous mutants with intact mexR and nalC genes were also identified, indicating that additional, yet uncharacterized resistance mechanisms may exist. Together, these findings demonstrate that resistance to QQ compounds may arise through different mechanisms and that these mutations may occur in a clinical setting, although their generality remains unclear [142].

Insights from the *P. aeruginosa* model and related studies [24] suggest that bacteria may evolve resistance to QS-targeting strategies through several potential mechanisms, including:

- Mutations in LuxR and LysR-like receptors that enhance their affinity for AIs thereby lowering the required AIs concentration threshold.
- Increased AIs production to ensure sufficient signal availability for QS activation.
- Synthesis of modified AIs to become less susceptible to different QSIs.

Testing these predictions using traditional microbiology methodologies is challenging. For example, quantifying resistance to QQ requires isolating resistant organisms from a mixed population. However, unlike conventional antibiotics—where only resistant mutants survive and are easily identified—both resistant and sensitive organisms may continue to grow in the presence of QQ molecules [144].

To overcome this obstacle, Beckmann et al. [144] used the Avida system to simulate the evolution of digital organisms [145]. These are self-replicating computer programmes subject to mutation and natural selection within a computational environment. Their results demonstrate that resistance to QQ can evolve, at least in silico. While signal-receiving-impaired mutants initially disrupted QS effectively, populations exposed to increasing levels of such mutants developed resistance over time. This occurred via a shift in quorum threshold, with organisms triggering quorum at lower population densities than the original strain. Notably, the highest resistance levels evolved under gradually increasing mutant introduction rates, indicating that sustained selective pressure can drive adaptive responses [144].

According to Anguige et al. [146] QQ treatment is only effective below a critical biofilm depth, making drug penetrability a key factor in the design of antivirulence compounds. Resistance to QQ in biofilms may therefore arise through reduced permeability, for example via overproduction of ECM components that sequester QQ agents, such as ndvB-encoded glucans known to bind aminoglycosides [147]. Other theoretical studies show that, for biofilms, the timing of QQ treatment is critical for effective prevention of QS-mediated virulence [148].

Defoirdt [149] reports that resistance to QS inhibition appears to spread more slowly during host infection than resistance to conventional antibiotics. However, the selective dynamics of QS are highly context-dependent, particularly regarding whether QS regulates “private goods” (benefitting only the producing cell) or “public goods” (benefitting the broader population). In vitro studies have demonstrated that resistance can spread when growth depends on QS-controlled private goods, but it may be constrained when public goods are involved, as “sensitive cheater” cells exploit resistant producers [149]. Crucially, recent in vivo experiments using *Vibrio campbellii* across multiple host infection cycles showed that resistance to QSI did not spread when initially rare. Although it could reach fixation when initially common, its spread was approximately tenfold slower than that of antibiotic resistance [150]. These findings suggest that while QSI is not evolution-proof, it exerts weaker selective pressure than conventional antibiotics during infection, supporting its potential as a more sustainable therapeutic strategy. Nonetheless, caution is warranted, particularly in pathogens harbouring broad-spectrum resistance mechanisms such as multidrug efflux pumps, which may confer cross-resistance to certain QSI compounds. Additional research across diverse pathogen-host systems, particularly in the presence of natural microbiota, is required to assess the generalizability of these results [151].

Although competition experiments between QS-deficient and QS-proficient strains have provided insight into the potential selection of QQ resistance, further studies are required, particularly those involving quenching agents under conditions that better mimic in vivo environments. Current QQ compounds present notable limitations: highly specific inhibitors often display modest activity, whereas more potent molecules may exhibit significant toxicity. In contrast, enzymes such as acylases and lactonases, which degrade homoserine lactone signals, appear both specific and effective, making them promising tools for experimental investigation [152].

Current evidence highlights QSIs as an important and conceptually attractive antivirulence approach, particularly in the broader effort to develop anti-infective therapies that do not rely exclusively on direct bactericidal or bacteriostatic activity. Because they target virulence regulation rather than growth itself, they are generally associated with lower selective pressure than conventional antibiotics, and resistance appears to spread more slowly and less uniformly. This gives QSIs particular significance as potential components of more sustainable therapeutic strategies. However, the true clinical implications of these observations remain incompletely understood, as available evidence is still limited by the small number of experimental systems, the predominance of model-organism data, and the context-dependent nature of QS regulation across infection settings.

### 5.2. Impact on Host Microbiota 

The human body is colonized by diverse microorganisms across its surfaces and in cavities connected to the external environment [153]. These permanent and transient inhabitants form complex interspecies interactions, collectively constituting the microbiome.

The microbiome is a dynamic community of microorganisms interacting closely with the host and environment, significantly influencing physiological processes, health outcomes, and treatment responses [154,155]. Advances in high-throughput sequencing technologies have enabled detailed characterization and functional profiles of these communities, providing deeper insight into their interactions with host organs and tissues [156].

In these microbial communities, QS facilitates both intra- and interspecies communication via signalling molecules known as AIs. This system orchestrates collective actions, such as biofilm development and pathogenic virulence. Crucially, these QS networks are not exclusive to pathogens; rather, they are pervasive throughout diverse microbial ecosystems, actively engaging both commensal and beneficial microorganisms [157].

Consequently, QQ strategies, while targeting pathogenic QS systems, may also interfere with communication within the host microbiota, although the effect of QQ on the microbiota is not yet fully known [157,158]. Such disruption could alter microbial community structure and function, potentially leading to dysbiosis and unintended clinical consequences [158]. Therefore, to develop effective therapeutic interventions, it is necessary to ensure that such therapies are target-specific, and not broad in their actions, which underscores the need for deeper understanding of QS regulation and interactions in specific environments [157].

A comprehensive understanding of the impact of QQ on the human microbiota remains limited by the lack of integrated data. This includes detailed knowledge of microbial composition, interspecies interactions, QS signalling molecules, and the functional effects of these communities on host health and surrounding tissues. Such complexity calls for molecule-specific investigation, making research in this area inherently long-term and resource-intensive [158].

## 6. Methods

A comprehensive literature search was conducted using databases such as PubMed, Google Scholar, and Scopus, focusing primarily on studies published between 2021 and 2026; however, earlier pivotal publications were also included when deemed relevant. Core search terms included combinations of: (“quorum sensing” OR “quorum quenching”) AND (“biofilm” OR “antimicrobial resistance”); (“quorum sensing” OR “quorum quenching”) AND (“Pseudomonas aeruginosa” OR “Staphylococcus aureus”); (“biofilm inhibition” AND “quorum sensing”) AND (“Pseudomonas aeruginosa” OR “Staphylococcus aureus”). While advanced search methods prioritized literature from the past five years, this review analyses research spanning from 1995 to 2026. Inclusion criteria: (1) peer-reviewed original research articles, (2) systematic and narrative reviews published in high-impact journals, (3) studies focusing on *P. aeruginosa* and *S. aureus* as model organisms, and (4) articles published in English. Exclusion criteria: (1) non-peer-reviewed reports, (2) conference abstracts without full-text availability, and (3) predatory or low-quality publications.

## 7. Conclusions and Final Research Perspectives

AMR continues to undermine the effectiveness of conventional antibiotics and represents one of the most pressing global health challenges, particularly in the context of chronic and biofilm-associated infections. In this context, strategies targeting bacterial virulence rather than viability have attracted increasing attention. This review critically evaluated the current state of QQ research, with a primary focus on *P. aeruginosa* and *S. aureus*, two clinically relevant biofilm-forming pathogens that serve as major models for studying QS-driven virulence and its therapeutic targeting. Table 7 provides a comparative summary of the principal QQ strategies, with emphasis on their potency, target specificity, stability, bioavailability, safety profile, cost, and potential for clinical translation.

QS plays a critical role in coordinating virulence and bacterial behaviours, including biofilm formation, toxin production, motility, and immune evasion. Interference with this communication system through QQ has emerged as a conceptually attractive anti-virulence approach. Unlike traditional antibiotics, QQ strategies aim to attenuate pathogenicity without directly inhibiting bacterial growth, potentially reducing selective pressure for resistance development. Synergistic regimens combining QQ agents with antibiotics, efflux pump inhibitors, or biofilm-disrupting compounds warrant systematic evaluation. In this context, QQ should not be viewed as a replacement for antibiotics but rather as an adjunctive strategy integrated into multimodal treatment protocols. AI and ML-based screening platforms may accelerate the identification of potent and selective QS inhibitors while reducing R&D costs. Rational drug design, supported by structural biology and molecular modelling, can facilitate the optimization of receptor-specific inhibitors with improved stability and minimized off-target effects. NP-based delivery systems offer additional advantages by enhancing targeted drug delivery, improving biofilm penetration, and enabling controlled release profiles. Enzyme-based QQ strategies and monoclonal antibodies directed against signalling molecules represent further innovative modalities that merit exploration.

Long-term experimental evolution studies are needed to assess whether and how bacteria adapt to sustained QS disruption. The majority of available data originate from simplified experimental systems that do not fully recapitulate the complexity of human infections. Biofilms formed in vivo are structurally heterogeneous, influenced by host immunity, nutrient availability, oxygen gradients, and polymicrobial interactions. Consequently, the therapeutic efficacy observed under controlled laboratory conditions may not directly translate to clinical benefit.

Several limitations must be addressed before QQ can be considered a viable therapeutic strategy. The pharmacokinetic and pharmacodynamic properties of many QQ agents remain poorly defined, particularly with respect to bioavailability, tissue penetration, metabolic stability, and dosing. These issues are especially relevant in conditions such as cystic fibrosis, where preventing biofilm formation could be used at early stage of infection. In addition, the long-term consequences of QS interference remain uncertain, as QQ may still promote adaptive responses, including compensatory mutations or activation of alternative virulence pathways, particularly in pathogens with complex QS networks such as *P. aeruginosa*.

Another important concern is the potential impact of QQ on the host microbiome. Because QS is also used by commensal bacteria, non-selective interference may disturb beneficial microbial communities. Furthermore, current regulatory frameworks are not well suited to anti-virulence therapies, as conventional clinical endpoints focus mainly on bacterial eradication or mortality rather than reductions in virulence, inflammation, or recurrence.

Standardization of in vivo biofilm models that more accurately mimic human disease is critical for generating reproducible and clinically relevant data. Pharmacokinetic analyses, toxicity assessments, and microbiome monitoring must become integral components of preclinical evaluation. Clinical translation will require carefully designed pilot trials in well-defined patient populations, such as individuals with chronic lung infections, IAIs, or complex wound biofilms. Additionally, interdisciplinary collaboration among microbiologists, clinicians, pharmacologists, bioengineers, and regulatory experts will be necessary to establish realistic development pathways for anti-virulence therapeutics.

AI and ML are transforming efforts to combat AMR by enabling the analysis of large-scale medical, pharmacological, and genomic datasets. These technologies support early prediction of resistant strains and facilitate the development of targeted therapeutic strategies. In clinical settings, AI-driven diagnostic tools improve the speed and accuracy of pathogen identification, thereby reducing antibiotic misuse and optimizing patient care pathways [160].

Beyond diagnostics, AI plays a critical role in accelerating the drug discovery pipeline, particularly through in silico screening of large chemical libraries. For example, a neural network-based study screened approximately 7500 compounds, leading to the identification of abaucin, a molecule effective against *Acinetobacter baumannii* infections [161]. Such approaches also enable the efficient screening of underutilized chemical space, including natural product databases. With over 300,000 compounds in SciFinder yet to be tested for antibacterial activity, computational methods allow rapid identification of candidates capable of targeting essential bacterial enzymes. Compared to traditional experimental techniques, these methods are more cost- and time-efficient, enabling large-scale analyses within days. However, their applicability remains limited to targets with available three-dimensional structural data in public databases [162]. Furthermore, AI- particularly through ML and deep learning techniques is instrumental not only in the design of novel antibiotics but also in enhancing the efficacy of existing drugs through the development of synergistic combinations [163]. Despite these advances, important challenges remain. The use of AI in healthcare raises ethical concerns related to the handling of sensitive genetic and clinical data, emphasizing the need for rigorous data collection and strict adherence to informed consent protocols [164].

An alternative strategy is drug repurposing, also referred to as drug reprofiling or repositioning. This development approach involves identifying novel pharmacological applications for already approved pharmaceuticals beyond their original intended use. By leveraging existing drugs, developers can bypass the initial stages of the drug discovery pipeline, leading to substantial savings in both time and costs. Furthermore, many pharmaceutical agents possess secondary mechanisms of action that remain partially characterized, enabling them to function effectively against various bacteria, either through direct antibacterial activity or by acting as inhibitors of bacterial virulence [80].

Taken together, the available evidence suggests that QQ are a potentially valuable supportive strategy, especially when combined with existing antimicrobial therapies. Its future clinical relevance will depend on further mechanistic studies, the use of standardized and clinically meaningful infection models, the development of more effective delivery systems, and careful evaluation of safety, including possible effects on the host microbiome. Well-designed translational and early clinical studies will also be necessary to determine whether the promising results observed so far can be translated into real therapeutic benefit. Ultimately, only a balanced and evidence-based approach that takes into account both the opportunities and the current limitations of QQ will show whether it can become a truly useful option in the treatment of biofilm-associated infections.

## Figures and Tables

**Table 1:** WHO Bacterial Priority Pathogens List, 2024 [5].

Priority Group	Organism	Resistance
Critical group	*Enterobacterales*	Carbapenem-resistant
	*Enterobacterales*	Third-generation cephalosporin-resistant
	*Acinetobacter baumannii*	Carbapenem-resistant
	*Mycobacterium tuberculosis*	Rifampicin-resistant
High group	*Salmonella* Typhi	Fluoroquinolone-resistant
	*Shigella* spp.	Fluoroquinolone-resistant
	*Enterococcus faecium*	Vancomycin-resistant
	*Pseudomonas aeruginosa*	Carbapenem-resistant
	Non-typhoidal *Salmonella*	Fluoroquinolone-resistant
	*Neisseria gonorrhoeae*	Third-generation cephalosporin- and/or fluoroquinolone-resistant
	*Staphylococcus aureus*	MRSA
Medium group	Group A *Streptococci*	Macrolide-resistant
	*Streptococcus pneumoniae*	Macrolide-resistant
	*Haemophilus influenzae*	Ampicillin-resistant
	Group B *Streptococci*	Penicillin-resistant

Abbreviations: WHO—World Health Organisation; MRSA—Methicillin-Resistant *Staphylococcus aureus*.

**Table 2:** Quorum sensing-controlled virulence factors in *P. aeruginosa* and their role in pathogenesis.

Category	QS-Regulated Factors	Main Function	Role in Pathogenicity	References
Toxins and proteases	ExoS, ExoT, ExoU, ExoY; LasA; LasB; proteases	Cytotoxicity, proteolysis, host–cell disruption	Tissue damage, immune evasion, enhanced virulence	[25,26]
Biofilm components	Exopolysaccharides, rhamnolipids	Biofilm formation and stabilization	Persistence, protection from host defences, reduced antibiotic susceptibility	[27]
Iron acquisition and secondary metabolites	PYO, pyoverdine	Iron scavenging, oxidative stress induction	Host damage, impaired immunity, improved bacterial survival	[28]
Secretion systems	T6SS linked to the *pvf* cluster	Delivery of virulence-associated effectors, bacterial competition	Host invasion and interbacterial competition	[29]
Regulatory network	Las, Rhl, Pqs, Iqs; ~70 transcription factors	Coordinated control of virulence gene expression	Dynamic adaptation of motility, biofilm maturation, and toxin production	[27]
Antibiotic resistance-associated traits	Biofilm formation, efflux pump activity	Reduced drug penetration and enhanced bacterial protection	Increased resistance and treatment difficulty	[30,31]

Abbreviations: QS—quorum sensing; ExoS—exoenzyme S; ExoT—exoenzyme T; ExoU—exoenzyme U; ExoY—exoenzyme Y; T6SS—type VI secretion system; pqs—Pseudomonas quinolone signal; pyf—pyoverdine-associated factor; Iqs—integrated quorum-sensing signal.

**Table 7:** Comparative overview of quorum-quenching (QQ) strategies: potency, specificity, stability, bioavailability, toxicity, cost, and translational potential.

QQ Strategy and Examples of Substances	Potency	Specificity	Stability	Bioavailability	Toxicity	Cost	Ease of Clinical Translation
Natural Compounds (e.g., sesamol, limonene, geraniol, piperine) [49,50,51,58,60,61,62,63,65]	The activity of several compounds is strongly influenced by environmental and methodological factors. Several compounds exhibit limited standalone activity and achieve optimal effects only in combination with antimicrobial agents.	These compounds may affect multiple pathways and are not always strictly QS-specific.	Prone to degradation, environmental sensitivity.	Poor solubility, rapid metabolism.	Preliminary safety may be favourable for some compounds, but toxicity remains insufficiently characterized.	Often widely available and inexpensive.	Limited in vivo data, lack of standardization, high variability across models. Many natural QQ agents function more effectively as antibiotic adjuvants rather than conventional therapeutics.
Natural Compounds with Antibiotic Combinations [49,52,64,65]	Often shows synergistic or additive effects with antibiotics.	Activity may involve both QS-related and non-QS mechanisms.	Moderate.	Combination therapy may improve overall efficacy, although intrinsic bioavailability often remains limited.	The safety profile depends on both the natural compound and the antibiotic used.	Moderate.	Promising due to well-known mechanisms of antibiotics, but needs clinical validation.
Synthetic Compounds [73,75,81,82,83]	Often associated with low IC_50_ values and potent QS-inhibitory activity.	Target specific QS systems such as LasR and PqsR.	Chemically optimized structures in silico, flexibility of synthetic design allows for structural optimization and improved selectivity.	Can be optimized, but often still limited.	Cytotoxicity can be evaluated in silico in the early stages of the study, but their safety, PK/PD, and in vivo efficacy remain insufficiently established for most candidates.	Moderate-to-high development costs.	Requires further in vivo validation and safety profiling.
Nanoparticles (e.g., SqNP, Co_3_O_4_ NPs) [55,74,79,86,95,159]	Often shows pronounced antibiofilm and QS-inhibitory activity, which may depend on particle size and shape.	Can target specific pathways or delivery sites.	Enhanced physiochemical stability.	Improved penetration through biofilms and tissues.	Potential off-target effects.	Production and formulation processes are complex and costly.	The lack of standards for the NPs, safety concerns.
Enzymatic QQ (e.g.,lactonases, acylases) [6,66,67,68,70,71,78]	Efficient degradation of QS signal molecules has been demonstrated in an experimental model.	Often relatively specific for signal molecules such as AHLs, although substrate range may vary between enzymes.	Enzyme stability and durability remain important formulation challenges.	In vivo delivery, retention, and protection from degradation remain challenging.	Enzymatic QQ is often considered potentially biocompatible, but safety data remain limited and enzyme-specific.	Production, purification, stabilization, and formulation may increase development costs.	Clinical translation is constrained mainly by stability, delivery, and formulation requirements.
Synthetic hybrids/Novel Mechanisms (e.g., c-di-GMP modulators G-quadruplex inducers) [76,77]	Promising antivirulence activity has been reported in early-stage studies.	Mechanistically targeted approaches have been proposed, although specificity remains compound-dependent.	Moderate	Moderate	Toxicity data remain limited and are available for only a subset of compounds.	Development costs are increased by design complexity, synthesis, and early-stage optimization requirements.	Low to moderate. Early stage research, limited validation.

Abbreviations: QS—quorum sensing; QQ—quorum quenching; AHL—N-acyl homoserine lactone; IC_50_—half-maximal inhibitory concentration; LasR—Las quorum-sensing transcriptional regulator; PqsR—Pseudomonas quinolone signal regulator; PK/PD—pharmacokinetics/pharmacodynamics.

## Data Availability

No new data were created or analyzed in this study.

## Abbreviations

The following abbreviations are used in this manuscript:

3OC12-HSL	N-(3-oxododecanoyl)-L-homoserine lactone
3OC6-HSL	N-(3-oxohexanoyl)-L-homoserine lactone
ABR	Antibiotic Resistance
AHQ	2-alkyl-4-(1*H*)-quinolone
AHL	N-acyl Homoserine Lactone
AI	Artificial Intelligence
AIs	Autoinducers
AIPs	Autoinducing Peptides
AMR	Antimicrobial Resistance
ASA	Acetylsalicylic Acid
BPPL	Bacterial Priority Pathogens List
C-30	4-bromo-5-(bromomethylene)-2(5*H*)-furanone
C4-AHL	N-butanoyl-L-homoserine lactone
CABHL	N-cyclohexanoyl-L-homoserine lactone
CADD	computer-aided drug design
*C. elegans*	*Caenorhabditis elegans*
CF	Cystic Fibrosis
CFTR	Cystic Fibrosis Transmembrane Conductance Regulator
CFU	Colony-forming units
COPD	Chronic Obstructive Pulmonary Disease
Co_3_O_4_	Cobalt(II,III) oxide
CSWIs	Chronic Surgical Wound Infections
CYP	Cytochrome P450
DINCC	N′-dodecyl-N-isonicotinyl chitosan chloride
DMDAC	N′,N′-dimethyl-N′-dodecyl-ammonium chloride-N-amino-acetyl chitosan
DMDBC	N′,N′-dimethyl-N′-dodecyl-ammonium chloride-N-benzoyl chitosan
DNA	Deoxyribonucleic Acid
eDNA	extracellular DNA
ECM	extracelullar matrix
EPS	Extracellular Matrix
ExoS	Exoenzyme S
ExoT	Exoenzyme T
ExoU	Exoenzyme U
ExoY	Exoenzyme Y
FABHL	N-(4-{4-fluoroanilino}butanoyl)-L-homoserine lactone
FDA	Food and Drug Administration
FRET	Fluorescence resonance energy transfer
FtsZ	Filamenting temperature-sensitive mutant Z protein
GLASS	Global Antimicrobial Resistance and Use Surveillance System
HCD	high-cell-density
HHQ	2-heptyl-4-(1H)-quinolone
IAIs	Implant-Associated Infections
IC50	Half maximal inhibitory concentration
IQS	Integrated QS Signal
IQR	Interquartile Range
LCD	low-cell-density
LPS	Lipopolysaccharide
LuxI	Autoinducer synthase (LuxI-type enzyme)
LuxR	Transcriptional regulator (LuxR-type receptor)
Mabs	Monoclonal Antibodies
MDR	Multidrug Resistance
MDR PA	Multi Drug Resistant *Pseudomonas aeruginosa*
MIC	Minimum Inhibitory Concentration
ML	Machine Learning
MRSA	Methicillin-Resistant *Staphylococcus aureus*
NPs	Nanoparticles
NTM	Non-Tuberculous Mycobacteria
*P. aeruginosa*	*Pseudomonas aeruginosa*
PQS	Pseudomonas Quinolone Signal
PYO	Pyocyanin
pwCF	people with Cystic Fibrosis
QQ	Quorum Quenching
QS	Quorum Sensing
QSI	Quorum Sensing Inhibitors
R&D	Research and Development
RIP	RNAIII-inhibiting peptide
ROS	Reactive Oxygen Species
RR	Rifampicin-Resistant
*S. aureus*	*Staphylococcus aureus*
SD	Standard Deviation
SpA	Staphylococcal protein A
SqNP	Qualenyl hydrogen sulfate nanoparticle
SrtA	Sortase A
SSIs	Surgical Site Infections
VRSA	Vancomycin resistant *Staphylococcus aureus*
TACN	1,4,7-triazacyclononane
T6SS	Type VI secretion system
Ti0_2_	Titanium dioxide
WHO	World Health Organization
ZnO	Zinc Oxide
